# Independent and combined effects of environmental factors and *CYP2C19* polymorphisms on the risk of esophageal squamous cell carcinoma in Fujian Province of China

**DOI:** 10.1186/s12881-015-0156-3

**Published:** 2015-03-17

**Authors:** Xian-E Peng, Hua-Fang Chen, Zhi-Jian Hu, Xi-Shun Shi

**Affiliations:** Department of Epidemiology and Health Statistics, School of Public Health, Fujian Medical University, 88 Jiaotong Road, Fuzhou, 350004 China; Key Laboratory of Ministry of Education for Gastrointestinal Cancer, Research Center of Molecular Medicine, Fujian Medical University, Fujian, China; CDC of XiaMen, 681-685Shengguang Road, Xiamen, China

**Keywords:** Case–control study, Risk factors, Genotype, Gene–environment interaction

## Abstract

**Background:**

The purpose of this study was to explore the effects of *CYP2C19* gene polymorphisms and various environmental factors and their interactions on the risk of esophageal squamous cell carcinoma (ESCC) in a Chinese Han population.

**Methods:**

A 1:2 frequency-matched case control study of 285 patients and 570 controls was conducted from June 2010 to May 2011 in AnXi of Fujian province, China. Environmental factors were investigated using a self-administered questionnaire and genotypes were determined using polymerase chain reaction restriction fragment length polymorphism based methods. Unconditional logistic regression models were used for statistical evaluation.

**Results:**

Current or former smoking, consumption of pickled vegetables or hot beverages/food, having a first degree relative with ESCC and history of reflux esophagitis were significantly associated with increased ESCC risk, whereas tea drinking and consumption of fresh vegetables and fruits were significantly associated with decreased risk. The CYP2C19*2 GA/AA genotype was significantly more prevalent in ESCC patients and individuals with at least one copy of the CYP2C19*2 A allele had a 3.19-fold increased risk (adjusted 95% confidence interval (CI): 2.21–4.61, *P* < 0.001) of ESCC compared with those without this allele. We found no significant associations between CYP2C19*3 genotypes and ESCC. The Cyp2C19*2 polymorphism appeared to have a multiplicative joint effect with tea drinking and hot beverage/food consumption (gene–tea drinking: *P*_interaction_ = 0.042; hot beverage/food consumption: *P*_interaction_ = 6.98 × 10^−6^) and an additive joint effect with pickled vegetable consumption (interaction contrast ratio = 1.96, 95% CI: 0.12–3.80).

**Conclusions:**

Our findings suggest that the CYP2C19*2 polymorphism plays an important role in the development of ESCC in the Chinese population, modified by tea drinking and consumption of pickled vegetables or hot beverages/food. Further studies are warranted to confirm our results.

**Electronic supplementary material:**

The online version of this article (doi:10.1186/s12881-015-0156-3) contains supplementary material, which is available to authorized users.

## Background

Esophageal squamous cell carcinoma (ESCC), a treatment resistant cancer that can withstand a combination of surgery, chemotherapy and radiotherapy [1], is the fourth most commonly diagnosed cancer and the fourth leading cause of cancer death in China. ESCC occurs through a complex multistage process that may involve a combination of carcinogen exposure and genetic susceptibility. Although smoking, alcohol drinking and prolonged use of wood or charcoal as sources of fuel for cooking and heating (resulting in excessive smoke inhalation) have been demonstrated as lifestyle factors that contribute to the development of the disease [2-4], the DNA sequence variations that confer an additional risk remain largely unknown.

Cytochrome P450 2C19 (CYP2C19) is an important phase I enzyme expressed abundantly in endothelial and smooth muscle cells [5,6]. This enzyme is involved in the metabolism of numerous therapeutic drugs and other xenobiotics, including S-mephenytoin, omeprazole, diazepam, proguanil, propranolol and certain antidepressants [7]. CYP2C19 is also known to be involved in the detoxification or inactivation of potential carcinogens [8] and the bioactivation of certain environmental procarcinogens to produce reactive DNA binding metabolites [9-11]. Therefore, it is conceivable that *CYP2C19* gene polymorphisms may play a major role in inter-individual variability in drug response, drug–drug and drug–xenobiotic interactions and susceptibility to chemical-induced diseases.

Several polymorphisms of *CYP2C19* are known to be associated with reduced enzyme activity; however, most cases are due to either CYP2C19*2, which is characterized by a point mutation in exon 5 (681G → A, rs4244285), or CYP2C19*3, which has a mutation in exon 4 (636G → A, rs4986893) [12]. The presence of these alleles is considered to be sufficiently predictive for the phenotypes to be inferred from them. Thus, individuals homozygous for CYP2C19*2 or CYP2C19*3 are considered poor metabolizers (PMs), whereas those with at least one CYP2C19*1 allele are classified as extensive metabolizers (EMs) [13]. Despite the biological plausibility of *CYP2C19* functional polymorphisms as modulators of susceptibility to cancer, inconsistent findings have appeared in the literature; for example, CYP2C19 PMs have a high incidence of stomach cancer and lung cancer [14], but conversely have a low incidence of bladder cancer [13]. Furthermore, the role of these gene variants in ESCC has not been sufficiently well investigated. Normally, Asian people have a higher incidence of CYP2C19*3 than Caucasians [15,16] and the two mutations CYP2C19*2 and CYP2C19*3 have been shown to account for almost 100% of Asian PM alleles. Therefore, it would be interesting to determine whether the high incidence of ESCC is correlated with a greater frequency of CYP2C19*3 or CYP2C19*2 in the Chinese population.

In the present study, we hypothesized that individuals with the CYP2C19*2 or CYP2C19*3 polymorphism may have a higher risk of ESCC. We also speculated that there should be a synergistic interaction between the effect of environmental factors and that of the genetic variation in the risk of ESCC.

## Methods

### Ethics statement

This study was approved by the institutional ethical committees of Fujian Medical University. Written informed consent was obtained from all participants before their participation in the study. All investigations performed in this study were conducted in accordance with the guidelines of the 1975 Declaration of Helsinki.

### Study population

A 1:2 frequency-matched case control study was performed between June 2010 and May 2011 in AnXi, in the Fujian Province of China. Two hundred and eighty-five patients (168 males and 117 females; mean age 59.67 ± 9.83 years) were diagnosed with histologically confirmed ESCC at AnXi Hospital. Five hundred and seventy cancer-free control subjects (336 males and 234 females; mean age 59.71 ± 9.84 years) were chosen randomly during the same period from among local residents who underwent a routine health check and were free from any known major diseases. Controls were frequency matched with ESCC patients according to sex, age (within 5 years), ethnicity and area of residence. All subjects were genetically unrelated ethnic Han Chinese from AnXi or the surrounding regions.

### Data collection

A standard questionnaire was administered to cases and controls by specially trained interviewers. Questions covered demographic characteristics (e.g. age, education level, job, marital status, sex), dietary habits, lifestyle habits such as tobacco smoking and alcohol drinking, personal medical history and family history of cancer. Non-smokers were defined as individuals who had never smoked cigarettes or who had smoked less than 100 cigarettes in lifetime. Ever tea drinking was defined as drinking at least 1 cup of green tea per week for more than 6 months. Ever alcohol drinkers were subjects who had consumed any alcoholic beverage, including beer, wine or distilled spirits, at least once per week for a minimum of 6 months.

### Genetic polymorphism genotyping

Blood samples were collected with a standard venepuncture technique and ethylenediaminetetraacetic acid-containing tubes. DNA was extracted from the blood cell pellet using a Blood Genome DNA Extraction Kit (Takara Bio Inc., Ōtsu, Japan) and stored at −20°C. CYP2C19*2 (681G → A, rs4244285) and CYP2C19*3 (636G → A, rs4986893) genotypes were determined using polymerase chain reaction (PCR)-based restriction fragment length polymorphism. PCR was performed with 25 μl of reaction mixture containing 100 ng of DNA, 0.1 mmol/l of each primer, 0.2 mmol/l of deoxynucleoside triphosphate, 1.0 U of Taq DNA polymerase (Takara Bio Inc.), 1 × reaction buffer and 1.5 mmol/l of MgCl_2_. The PCR profile comprised an initial melting step for 2 min at 95°C, followed by 35 cycles of 30 s at 94°C, 30 s at 58°C and 30 s at 72°C and a final elongation step of 10 min at 72°C. The forward primer 5′-AATTACAACCAGAGCTTGGC-3′ and the reverse primer 5′-TATCACTTTCCATAAAAGCAAG-3′ were used to detect the CYP2C19*2 allele and the PCR products digested with SmaI I (New England Biolabs, Ipswich, MA); the 168 bp PCR products were cut into 117 and 51 bp fragments in the wild type (WT) but not in CYP2C19*2. The forward primer 5′-AAATTGTTTCCAATCATTTAGCT-3′ and the reverse primer 5′-ACTTCAGGGCTTGGTCAATA-3′ were used to detect the CYP2C19*3 allele and the PCR products digested with BamHI (New England Biolabs); the 271 bp PCR products were cut into 175 and 96 bp fragments in the WT but not in CYP2C19*3. The digested PCR products were analyzed on 3% agarose gels and stained with ethidium bromide. Individuals who inherit two mutant *CYP2C19* alleles, whether of the same type (*2/*2, *3/*3) or one of each (*2/*3) have a reduced capacity to metabolize CYP2C19 substrates and are considered to be PMs. Individuals who are homozygous (*1/*1) or heterozygous (*1/*2, *1/*3) for the WT CYP2C19*1 allele have an effective enzyme for metabolizing CYP2C19 substrates and are EMs. For quality control, genotyping was performed by laboratory personnel blinded to the case control status and blank controls were included in each plate. In addition, 10% of the samples were randomly selected and genotyped a second time; the concordance was 100%.

### Statistical analysis

Differences in the distribution of demographic characteristics and other risk factors for ESCC between patients and controls were tested using the chi-square test for category variables and Student’s *t*-test for continuous variables. Adjusted odds ratios (ORs) and their 95% confidence intervals (CIs) were calculated to evaluate the associations of lifestyle habits including smoking, alcohol/tea drinking and diet with risk of ESCC. All models were adjusted for age as a continuous variable, sex, education, income, occupation (farmer/other), family history of cancer in first degree relatives and other potential confounding factors. *P* values for OR trends were calculated using the order of each risk factor category as a categorical variable.

A goodness of fit chi-square test was used to assess whether the genotype distribution of *CYP2C19* polymorphisms was in Hardy–Weinberg equilibrium among the control subjects. Associations between *CYP2C19* genotypes and ESCC risk were evaluated using unconditional logistic regression with adjustment for potential confounding factors including age, sex, education, income, occupation, family history of cancer, consumption of vegetables, fruits and meat, smoking status (no/yes), alcohol drinking (no/yes) and tea drinking (no/yes).

Stratified analysis was used to explore potential gene–environment interactions. We dichotomized the genetic polymorphisms by grouping subjects into carriers and non-carriers of the risk genotype. Similarly, environmental factors were dichotomized by appropriate grouping. *P* values for multiplicative interactions were derived from a cross-product term for gene and environmental exposure introduced into a multiplicative model. Interaction contrast ratio (ICR) was used to evaluate potential additive interactions, as follows: ICR = OReg – ORe – ORg + 1, where OReg is the OR for both the genotype and the environmental exposure, ORe is the OR for the environment only and ORg is the OR for the genotype only. For ICR > 0, we concluded that there was a positive additive interaction. Ninety-five percent CIs for ICRs were calculated according to the method of Hosmer [17]; an ICR was considered to be statistically significant at an alpha level 0.05 if its 95% CI did not include zero.

All statistical analyses were performed using R software (version 2.14.1; The R Foundation, Vienna, Austria). Two-sided *P* values of < 0.05 were considered statistically significant.

## Results

### Characteristics of the study population

Associations between demographic characteristics and ESCC are shown in Table 1. There were no statistically significant differences between ESCC patients and controls in terms of median age, sex or marital status (all *P* > 0.05), indicating that the frequency matching was adequate. However, a greater proportion of farmers and a lower education level were observed among the patients compared with the controls (both *P* < 0.001).

**Table 1:** **ESCC risk factors and ESCC risk in a Chinese population**

**Characteristics**	**Cases**		**Controls**		***χ*** ^**2**^	***P*** **value**
	**No**	**%**	**No**	**%**		
Sex (Male)					0.00	1.000
Male	168	58.95	336	58.95		
Female	117	41.05	234	41.05		
Age group (years)					0.24	0.878
<50	97	34.02	191	33.51		
≥50	188	65.96	379	66.49		
Education level					13.62	0.001
Below middle school	146	51.23	223	39.12		
middle school	105	36.84	284	49.82		
High school or higher	34	11.93	63	11.05		
Marital status					2.02	0.365
Marriage	259	90.88	518	90.88		
Single	26	9.12	52	9.12		
Job					22.54	2.5 × 10^-6^
Farmer	235	82.46	382	67.02		
Non-farmer	50	17.54	188	32.98		
Household income, RMB/Month					0.71	0.339
<1000	180	63.16	243	42.63		
>1000	105	36.84	227	39.82		

### Associations between risk factors and ESCC risk

Table 2 shows adjusted associations between risk factors and risk of ESCC. Increased ESCC risk was associated with smoking, number of pack-years of cigarettes smoked, consumption of pickled vegetables, consumption of hot beverages/food, having a first degree relative with ESCC and having a history of reflux esophagitis. Decreased risk of ESCC was associated with tea drinking and consumption of fresh vegetables and fruits. Thus, a total of six environmental factors were selected for evaluation of their interactions with genetic variants in the risk of ESCC.

**Table 2:** **Association between lifestyle and dietary habits and ESCC risk**

**Variables**	**Cases**		**Controls**		***P*** ^**a**^	**Adjusted OR**
	**No**	**%**	**No**	**%**		**(95% CI)** ^**b**^
Smoking^1^					0.042	
No	130	45.61	281	49.30		Reference
Yes	155	54.39	289	50.70	0.194	2.02 (1.10 ~ 3.71)
Pack-years of smoking^1^						
Non-smoker	130	45.61	281	49.30		Reference
<30	56	19.65	124	21.75	0.814	1.54 (0.80 ~ 2.94)
>30	99	34.74	165	28.95	0.034	1.97 (1.05 ~ 3.68)
Alcohol drinking^2^					0.013	
No	220	77.19	451	79.12		Reference
Yes	65	22.81	119	20.88		1.09 (0.70 ~ 1.57)
Tea drinking^3^					2.99 × 10^-9^	
No	156	54.74	277	48.60		
Yes	129	45.26	293	51.40		0.63 (0.45 ~ 0.91)
Hot beverage/food intake^4^					5.74 × 10^-5^	
No	111	38.95	359	62.98		Reference
Yes	174	61.05	211	37.02		2.95 (2.06~4.22)
Pickled vegetables^5^						
No	135	47.37	355	62.28	5.47 × 10^-5^	Reference
Yes	150	52.63	215	37.72		1.87 (1.38~2.55)
Fresh vegetables and fruits (g/day)^6^					0.341	
<400	277	97.19	507	88.95		Reference
≥400	8	2.81	63	11.05		0.20 (0.10 ~ 0.44)
Meat (g/day)^7^					0.016	
<200	267	93.68	507	88.95		Reference
≥200	18	6.32	18	3.16		0.75 (0.42 ~ 1.35)
Family history of ESCC^8^					0.025	
No	241	84.56	523	91.75		Reference
Yes	44	15.44	47	8.25		1.77 (1.11 ~ 2.80)
History of reflux esophagitis^9^				0.026		
No	274	96.14	562	98.60		Reference
Yes	11	3.86	8	1.40		2.96 (1.14 ~ 7.67)

^a^ *P* value based on the Wald test.

^b^Odds ratio (*OR*) was determined using logistic regression and age, sex, education, income, marital status, tea drinking, alcohol drinking, smoking, pickled vegetables, fresh vegetables and fruits, meat, family history of ESCC, history of reflux esophagitis and hot beverage/food intake were included in the multivariate models.

### Association between *CYP2C19* polymorphisms and ESCC risk

Single nucleotide polymorphism (SNP) data, observed allele frequencies and Hardy–Weinberg test results are presented in Table 3. In the control group, the frequencies of the CYP2C19*2 A and CYP2C19*3 A alleles were 0.335 and 0.054, respectively, and were in Hardy–Weinberg equilibrium (both *P* > 0.05). On single allelic analysis, tests for association between ESSC and the two SNPs showed a significant difference only for CYP2C19*2 (G681A); the association remained significant after multiple comparison correction by permutation tests (*P* < 0.05; Table 3). The findings for each of the two SNP genotypes according to risk of ESCC are shown in Table 4 along with their adjusted ORs. The frequencies of the CYP2C19*2 GG, GA and AA genotypes in the ESCC patients differed significantly from those in the control group (*χ*^2^ = 43.56, *P* < 0.001, degrees of freedom = 2), with GA and AA being more frequent in patients than in controls. The homozygous mutant genotype (AA) of CYP2C19*3 was not detected in any subject, patient or control, and the frequencies of the CYP2C19*3 GG and GA genotypes did not differ significantly between the controls and patients.

**Table 3:** **SNPs identified for** ***CYP2C19***

**Nomenclature**	**SNP**	**Nucleotide changes**	**Effect**	**MAF** ^**a**^				***P*** **value for HWF in Controls**
				**NCBI** ^**b**^	**Controls**	**Cases**	***P*** **value for association analysis** ^**c**^	
CYP2C19*2 (exon5)	rs4244285	G681A	Splicing defect	0.256	0.335	0.488	2.31 × 10^-5^	**0.09**
CYP2C19*3 (exon4)	rs4986893	G636A	W212X	0.058	0.054	0.040	0.053	**0.177**

*HWE*: Hardy–Weinberg equilibrium; *MAF*: minor allele frequency.

^a^Major/minor allele.

^b^MAF for Chinese in the NCBI dbSNPs database. (http://www.ncbi.nlm.nih.gov/SNP).

^c^After correcting for multiple testing by Haploview software using 1000 permutations.

**Table 4:** **Association between** ***CYP2C19*** **genotype and risk of ESCC**

**Polymorphism. ID no**	**Genotypes**	**Cases n (%)**	**Controls n (%)**	**Crude OR (95% CI)**	**Adjusted OR (95% CI** ^**a**^ **)**	***P*** **-value** ^**a,b**^
CYP2C19*2						
rs4244285	GG	59 (20.70)	243 (42.63)	1.00 (Reference)	1.00 (Reference)	6.47 × 10^-8^
	GA	174 (61.05)	272 (47.72)	2.64 (1.87 ~ 3.71)	2.91 (1.99 ~ 4.25)	4.46 × 10^-7^
	AA	52 (18.25)	55 (9.65)	3.89 (2.42 ~ 6.26)	4.82 (2.84 ~ 8.17)	1.21 × 10^-6^
	GA + AA	226 (79.30)	327 (57.37)	2.85 (2.04 ~ 3.96)	3.19 (2.21 ~ 4.61)	2.16 × 10^-8^
*Test* for trend				2.08 (1.66 ~ 2.61)	2.30 (1.79 ~ 2.97)	3.17 × 10^-8^
CYP2C19*3						
rs4986893	GG	262 (91.93)	509 (89.30)	1.00 (Reference)	1.00 (Reference)	0.236
	GA	23 (8.07)	61 (10.70)	0.73 (0.44 ~ 1.21)	0.72 (0.422 ~ 1.24)	
EMs	*2/*2,*2/*3	226 (79.30)	491 (86.14)	1.00 (Reference)	1.00 (Reference)	0.009
PMs	*1/*1, *1/*2, *1/*3	59 (20.70)	79 (13.86)	1.62 (1.12 ~ 2.35)	1.71 (1.14 ~ 2.57)	

*n*: number of individuals; *OR*, odds ratio; *CI*: confidence interval.

^a^Adjusted for sex, age, education, income, smoking, tea drinking, body mass index and other variables.

^b^ *P* value based on the Wald test.

Unconditional logistic regression analysis was used to evaluate associations between the genotypes of the two *CYP2C19* polymorphisms and risk of ESCC. After adjustment for age, sex, income, marital status, education, smoking, tea drinking and other variables, a significant risk effect for ESCC was found to be associated with the CYP2C19*2 genotype. Specifically, compared with homozygous GG subjects, carriers of the heterozygous GA and homozygous AA genotypes had a significantly increased risk of ESCC (adjusted OR = 2.91, 95% CI: 1.99–4.25 for GA; adjusted OR = 4.82, 95% CI: 2.84–8.17 for AA) in an allelic dose–response manner (adjusted *P*_Trend_ = 0.000). On pooled analysis, we found that individuals with at least one copy of the CYP2C19*2 A allele had a 3.19-fold increased risk (adjusted 95% CI: 2.21–4.61, *P* < 0.001) of ESCC compared with those without this allele. We found no significant associations between the CYP2C19*3 genotype and ESCC in multivariate logistic regression models.

The association between being a CYP2C19 PM and having ESCC was further analyzed. The frequency of CYP2C19 PMs (the genotypes *2/*2 and *2/*3 but not *3/*3 were found in this study) was significantly higher among the ESCC patients than in the control group (20.70% vs 13.86%). After adjustment for age, sex, income, marital status, education, smoking, tea drinking and other variables, CYP2C19 PMs had a 1.71-fold increased risk of ESCC compared with EMs.

### Possible interactions between *CYP2C19* polymorphisms and environmental factors in ESCC risk

We also explored the combined effects of the CYP2C19*2 polymorphism and certain environmental factors on the risk of ESCC (Table 5). No significant interactions (multiplicative or additive) between CYP2C19*2 and alcohol drinking, smoking or consumption of fresh vegetables and fruits were observed. However, the CYP2C19*2 polymorphism appeared to have a multiplicative joint effect with tea drinking and hot beverage/food consumption (gene–tea drinking: *P*_interaction_ = 0.042; hot beverage/food consumption: *P*_interaction_ = 6.98 × 10^−6^) and an additive joint effect with pickled vegetable consumption (ICR = 1.96, 95% CI: 0.12–3.80). Specifically, tea drinking may decrease the effect of the GA/AA genotype on ESCC risk. By contrast, consumption of hot beverages/food or pickled vegetables may increase the risk effect of the GA/AA genotype.

**Table 5:** **Combined effects of** ***CYP2C19*** **polymorphism and environmental factors**

**Exposure**	CYP2C19*2	**Patients** ***n*** **(%)**	**Controls** ***n*** **(%)**	**Adjusted OR (95% CI)** ^**a**^	***P*** **value for multiplicative interaction** ^**a**^	**ICR (95% CI)** ^**a**^
Tea drinking					0.04	-1.77 (-3.92 ~ 0.38)
Never	GG	25 (8.77)	136 (23.86)	Reference		
Never	GA/AA	104 (36.49)	157 (27.54)	4.17 (2.46 ~ 7.08)		
Ever	GG	34 (11.93)	107 (18.77)	0.52 (0.32 ~ 0.84)		
Ever	GA/AA	122 (42.81)	170 (29.82)	1.92 (1.41 ~ 2.63)		
Alcohol drinking					0.051	-1.79 (-4.04 ~ 0.46)
Never	GG	40 (14.04)	197 (34.56)	Reference		
Never	GA/AA	180 (63.16)	254 (44.56)	3.63 (2.39 ~ 5.51)		
Ever	GG	19 (6.67)	46 (8.07)	2.32 (1.15 ~ 4.71)		
Ever	GA/AA	46 (16.14)	73 (12.81)	2.86 (1.60 ~ 5.10)		
Smoking					0.48	1.180 (-1.21 ~ 3.57)
Never	GG	30 (10.53)	134 (23.51)	Reference		
Never	GA/AA	100 (35.09)	147 (25.79)	3.41 (2.09 ~ 5.57)		
Ever	GG	29 (10.18)	109 (19.12)	2.23 (1.01 ~ 4.94)		
Ever	GA/AA	126 (44.21)	180 (31.58)	5.82 (2.80 ~ 12.09)		
Intake of fresh fruits and vegetables (g/day)					0.10	1.92 (-5.15 ~ 1.31)
<400	GG	55 (19.30)	212 (37.19)	Reference		
<400	GA/AA	222 (77.89)	295 (51.75)	2.94 (2.04 ~ 4.24)		
≥400	GG	4 (1.40)	31 (5.44)	0.41 (0.134 ~ 1.27)		
≥400	GA/AA	4 (1.40)	32 (5.61)	0.43 (0.14 ~ 1.33)		
Pickled vegetables					0.12	1.96 (0.12 ~ 3.80)
Never	GG	32 (11.23)	150 (26.32)	Reference		
Never	GA/AA	103 (36.14)	205 (35.96)	2.55 (1.61 ~ 4.06)		
Ever	GG	27 (9.47)	93 (16.32)	1.66 (0.90 ~ 3.04)		
Ever	GA/AA	123 (43.16)	122 (21.40)	5.17 (3.21 ~ 8.33)		
Hot Beverage and food intake					6.98 × 10^-6^	-0.66 (-6.42 ~ 5.10)
Never	GG	12 (4.21)	167 (29.30)	Reference		
Never	GA/AA	99 (34.74)	192 (33.68)	7.64 (3.90 ~ 14.94)		
Ever	GG	47 (16.49)	76 (13.33)	9.87 (4.67 ~ 20.85)		
Ever	GA/AA	127 (44.56)	135 (23.68)	15.85 (7.78 ~ 32.30)		

^a^Adjusted for age, sex, education, income, job, marital status, family history of cancer in first degree relatives and other potential confounding factors.

## Discussion

ESCC is prevalent among the Chinese population, with marked regional variations in incidence and mortality. Although the pathogenesis of ESCC is not fully elucidated, accumulative epidemiologic evidence has shown that genetic and environmental factors play crucial roles in its etiology. In the present study, we conducted a case control study to examine the role of the two most common functional variants of the *CYP2C19* gene (CYP2C19*2 and CYP2C19*3) in the development of ESCC in a Chinese population, including the effects of environmental risk factors. We found CYP2C19*2 to be the more common locus in the Chinese population, and multivariable logistic analysis revealed that the presence of the CYP2C19*2 A allele (AA or AG genotype) increased the risk of ESCC*.* Most of the established risk factors for ESCC evaluated in the present study had strong associations with ESCC risk. Generally in case control studies, a potential gene–environment interaction is assessed; our results show that the association between the CYP2C19*2 A variant and ESCC was modified by tea drinking and consumption of pickled vegetables or hot beverages/food. These findings suggest that CYP2C19 is involved in the detoxification of certain carcinogens involved in the development of ESCC. To our knowledge, this is the first study to report this gene–environment interaction between environmental factors and the CYP2C19*2 polymorphism with respect to the risk of ESCC in a Chinese population.

CYP2C19, one of the most important cytochrome P450s, is a key enzyme that is not only responsible for the metabolism of numerous therapeutic drugs [17-20] but is also suspected to play a major role in the detoxification or inactivation of potential carcinogens and the bioactivation of certain environmental procarcinogens to produce toxic DNA binding metabolites [8,21]. Therefore, CYP2C19 is considered an important defense against cancer. The human *CYP2C19* gene is highly polymorphic; the most important alleles are CYP2C19*2 (681G → A, rs4244285) and CYP2C19*3 (636G → A, rs4986893). The nucleotide changes in CYP2C19*2 and *3 lead to a splicing defect and a stop codon, respectively, and thereby to nonfunctional proteins and the PM phenotype [22]. The two main enzyme-deficient alleles of *CYP2C19* are suspected to be associated with susceptibility to cancer, though several studies concerning *CYP2C19* polymorphism and cancer susceptibility among various populations have reported inconsistent results [23-26]. In the present study, we found the frequency of CYP2C19*2 to be higher than that of CYP2C19*3 in Chinese Han subjects and the PM genotype was associated with an increased risk of ESCC. This finding is consistent with a recent meta-analysis by Wang et al. [14], who found an association between the *CYP2C19* PM genotype and increased risk of esophageal cancer (PM vs EM: OR = 2.93, 95% CI: 2.06–4.17) among 308 cases and 644 controls. However, our single-locus analysis found a significantly elevated risk of ESCC for the CYP2C19*2 allele but not for CYP2C19*3. Subjects carrying the CYP2C19*2 A allele (AA or GA genotype) had a higher risk of ESCC than GG and patients carrying at least one variant allele had a 3.19-fold increased risk of developing ESCC. CYP2C19*3 has a premature stop codon in exon 4 and it is biologically plausible that the CYP2C19*3 polymorphism is a modulator of cancer susceptibility; however, we did not find CYP2C19*3 to be associated with ESCC susceptibility in our study population. This finding is inconsistent with a recent report from Shi et al. [27], who found that the frequency of the CYP2C19*3 A allele was significantly higher in ESCC patients than in controls (5.57% vs 1.86%, *P* = 0.004). Our current understanding of carcinogenesis indicates a multifactorial and multistep process involving various genetic alterations and environmental factors and it is unlikely that risk factors for cancer act in isolation from each other. Therefore, differences in certain environmental factors may have contributed to this discrepancy. For example, Shi et al. [27] found that alcohol drinking was a risk factor for ESCC, whereas in the present study we did not find alcohol to be associated with ESCC. More importantly, the genetic effects of a SNP may depend on its interactions with environmental factors. Therefore, additional studies with a much larger sample size are warranted to confirm our results. Despite much investigation, the details of the role of environmental factors and pathogenic mechanisms in cancer remain a matter of speculation.

*CYP2C19* polymorphism is considered to be one of the factors that determine an individual’s susceptibility cancer through variations in ability to detoxify carcinogens and/or activate procarcinogens [28,29]. CYP2C19 PM status interacts significantly with environmental risk factors in modifying susceptibility to squamous cell carcinoma of the head and neck [30]. To explore the gene–environment interaction in ESCC, we investigated the effects of the *CYP2C192* gene and environmental factors on ESCC risk. Our findings suggest that the CYP2C19*2 polymorphism has a multiplicative joint effect with tea drinking and hot beverage/food consumption and an additive joint effect with pickled vegetable consumption. Specifically, consumption of hot beverages/food or pickled vegetables may increase the risk effect of the GA/AA genotype in ESCC. By contrast, tea drinking may decrease the risk effect of the GA/AA genotype.

Ecologic studies have shown higher risks of esophageal and gastric cancers in areas where the consumption of pickled food is high [31,32]. Consistent with this, our data show that pickled food consumption was associated with a 1.76-fold increased risk of ESCC compared with subjects who did not consume pickled food, and this association was modified by the presence of CYP2C19*2. In individuals carrying the CYP2C19*2 GA/AA genotype, the risk of ESCC in those who consumed picked vegetables was almost twofold greater than that of those who did not. The mechanisms by which pickled vegetables and the CYP2C19*2 polymorphism interact to influence the development of ESCC are unknown; however, previous studies have shown that the traditional method of preparing pickles by packing moist vegetables in jars for weeks or months allows fermentation and growth of fungi and yeasts [33,34] and can potentially yield carcinogenic substances such as N-nitroso compounds and mycotoxins [35-37]. Furthermore, polymorphisms in CYP2C19 largely account for PM status and influence metabolism, particularly detoxification of carcinogens [38]. Therefore, synergism between the CYP2C19*2 polymorphism and pickled vegetable consumption may be expected.

Numerous experimental and clinical studies have suggested that drinking beverages at high temperatures is a cause of esophageal cancer [39,40]. More tumors were observed and the size of esophageal papillomas was increased at temperatures of 70°C and above in a previous experimental study [41]. Consistent with this, our data show that drinking beverages at high temperatures was a risk factor for ESCC. Notably, we found significant synergism between consumption of hot beverages/food and the CYP2C19*2 polymorphism. The mechanisms by which drinking beverages at high temperatures and the CYP2C19*2 polymorphism interact to influence the development of ESCC are unknown and additional studies are warranted to explain and confirm this preliminary evidence.

The results of the present study also suggest possible interactions between tea drinking and the CYP2C19*2 polymorphism. Among individuals with the CYP2C19*2 GA/AA genotype, the risk of ESCC in tea non-drinkers was almost twice that in tea drinkers. Polyphenols in tea possess potent antioxidant and anti-inflammatory properties and modulate several signaling pathways, and these biochemical features are responsible for tea’s anticancer properties [42,43]. Previous studies have reported that green tea protects against cancers caused by various environmental carcinogens [43-45]. CYP2C19 is involved in the metabolism of many carcinogens; therefore, our results suggest that consumption of tea might have a potential ESCC prevention benefit in individuals with the CYP2C19*2 GA/AA genotype. Intervention trials are needed to provide more convincing evidence.

The potential limitations of the present study should be considered. First, this was a hospital-based case control study and selection bias may exist because the control subjects were recruited from a healthy population undergoing an examination, which may not accurately represent a geographically matched population with exposure to similar environmental factors. However, the control subjects came from the same region as the patients and were sampled randomly, which may have reduced the selection bias. Second, recall bias is inevitable in case control studies. However, this would not affect the genotype data and is therefore of less concern in the study of gene–disease associations. Finally, the selected *CYP2C19* polymorphism is a single example that was previously reported to have potential functional significance. Further studies of additional SNPs of functional significance are warranted to identify the role of *CYP2C19* polymorphism and gene–environment interactions in esophageal carcinogenesis.

## Conclusions

In summary, our findings suggest that the CYP2C19*2 A allele (AA or AG genotype) plays an important role in the development of ESCC in the Chinese population. In addition, our results show that the association between the CYP2C19*2 A variant and ESCC was modified by tea drinking and consumption of pickled vegetables or hot beverages/food. Since our study has limited sample size, further studies in a large population are needed to confirm these findings.
